# Supplementary material for: Patterns of Intron Gain and Loss in Fungi
Source: PLoS Biol. 2004 Nov 30;2(12):e422. doi: 10.1371/journal.pbio.0020422 (PMC532390; doi:10.1371/journal.pbio.0020422)
Supplement: Table S1 — Also available at http://genes.mit.edu/NielsenEtAl/. (4.3 MB ZIP). [file pbio.0020422.st001.zip › NielsenEtAl/html/1394.html]

AN0802.1.NCU03137.1.MG01311.1.FG00866.1


```
 CLUSTAL W (1.82) Multiple Sequence Alignments - Introns Inserted


Sequence 1: NCU03137.1	872 aa
Sequence 2: MG01311.1	765 aa
Sequence 3: FG00866.1	784 aa
Sequence 4: AN0802.1	730 aa
Alignment Length: 921 aa
Number Identitical Residues: 338 aa
Alignment Score (without introns) 16229


MG01311.1 	MQYVRNLSDSVSTAWNSINPATLSGAIDVIVVEQEDG~ALLCSPFHVRFGKFSLLRPYEK
NCU03137.1	MQYVRSLGDSVSTAWNSINPATLSGAIDVIVVEQEDG~TLACSPFHVRFGKFSLLRPSEK
FG00866.1 	MQYVRGLSESVSTAWNSINPATLSGAIDVIVVEHEDG~SLVCSPFHVRFGKFSLLRPSDK
AN0802.1  	MQYVRSISGSVSKTWNSINPATLSGAIDVIVIEQEDG1TLACSPFHVRFGKFSLLRPYEK
          	*****.:. ***.:*****************:*:*** :* **************** :*

MG01311.1 	K~VEFRVNGVKQPYSMKLGEGGEAFFVFETTDNIPKSMQTSPIVSPASS----PPFEPQQ
NCU03137.1	K~VEFRVNGVKQDYAMKLGEGGEAFFVFETTDTIPQSMQTSPIVSPASS----PPLNADQ
FG00866.1 	K~VEFKVNGVKQSYSMKLGEGGEAFFVFETTDRIPQSLQTSPLVSPASSPASSPPLNAEQ
AN0802.1  	K0VEFKVNGVKQDYAMKLGEGGEAFFVFPTTDEIPESMQTSPLISPMAS----PKMQSEE
          	* ***:****** *:************* *** **:*:****::** :*    * ::.::

MG01311.1 	PG--LQDPEAFDLDGESRKSRSPGP-VRPQPAIIQPGLAQDG1LITPSSTSPNLSVHRLK
NCU03137.1	SGP-LQEPESLDLNSVEGKTRSSSFNHRPPPTAL---LARDD1PIK--------------
FG00866.1 	SDSGLQEPEAFELDASESKPR------RPPPAALYKKENDDG1LITPLSTSPELPSVRPV
AN0802.1  	NLSTLQEPEYLDLDHSKGANHP------PLVSRTARATSDLG1LVTPVSQSDEPPPERPA
          	  . **:** ::*:  .   :.      *  :         .  :.. : : . .     

MG01311.1 	SGEWGN--FEHRPHSDDILRQRARRPSSNL---------DVEDMPLDLANPSDRSQSPPP
NCU03137.1	-GEWS----PFRPHSDGLLRQSARRMPSDG---------STHEGSEEGDSHTERSRSPPP
FG00866.1 	SGDWSA--KITRPHSDDVLRPSARSRGLDG---------NSSADDEE-HFASERSASPPP
AN0802.1  	RNSFGADVKLDRAVSDGILPTGSHSRSTSTGDYSLQLQRPASASSNEDPDLIRRSQSPPP
          	 ..:. .    *. **.:*   ::    . .. : . .        :      ** ****

MG01311.1 	LTRGQALGRAVTLSRELAAVNIRTQVTDEGDLMLDMSDMKNNEEDAFRAEILARKVLSQE
NCU03137.1	LTAPEALQRAMNLSRELAAANIPNHISETGDLMLDMTAFKNNEEDAFRAEILARKILTEE
FG00866.1 	LSAGEALQRARTLSKGLSAASIPTKVTDSGDLMLDMTGFKGNEEDILRAEILARQILSEE
AN0802.1  	ISLKEAVSRAMSLSQKLSTSNIPSRVTDSGDLMLDMTGYKSNEEDALRAEVVARKILAEE
          	::  :*: ** .**: *:: .* .:::: *******:  *.**** :***::**::*::*

MG01311.1 	LDGNYDVGALIGMDEQANLWIYHSEEAKEAAMKKMMET-SFTNPTVA-IDAASDPGYHSD
NCU03137.1	LDGNYDIGALFGVDEHGNLWIYSSIEAKEAAMKRTMDP-LRSASHMGNIDAASDPGYQSD
FG00866.1 	LDGNYDIGALFGFDEQGNLWIYSSEEAKQAAMNKTIEASLQSHRRSTAADAISDPGYQSD
AN0802.1  	LEGNYDIGALIGADEHGNLWIYSSEEAKEAANRRATLNAMRPGSAMS-ENAISDPGYHSD
          	*:****:***:* **:.***** * ***:** .:    :  .       :* *****:**

MG01311.1 	DSSGTAQT--PVHRRTESDVGQLALVQTPPSSPGST-TAGDPNRNYAKTLRLTSDQLKAM
NCU03137.1	ASDTSAATTLPIHRRADSDVGQMTL-QTPPSSPGSS-AAGDPNRNYAKTLRLTSDQLKAL
FG00866.1 	SSDATASP-MPLHRRTESDAGPMDLQTPPRTPPGSTRHSGDPNRNYAKTLRLTSQQLKDL
AN0802.1  	SDNSLRDPSFQMRHHRAKSDVQPGIPTPPQSPPNTA--PAEPVRSYAKTLRLTSDQLKAL
          	 ..    .:  ::::  ..     :  .* :.*.::  ..:* *.*********:*** :

MG01311.1 	NLKSGENTLSFTVNRATCSANMYLWKHETPVVISDIDGTITK~SDALGHVLNMIGRDWTH
NCU03137.1	NLKPGENSMSFTVNKATCQAYMFLWKHEVPVVISDIDGTITK~SDALGHVLNMIGRDWTH
FG00866.1 	ELKPGANSMAFTVNRATCKANMYLWRHETPVVISDIDGTITK~SDALGHVLNMIGRDWTH
AN0802.1  	KLKPGINDMSFSVNKATCTANMYLWRGDTPIVISDIDGTITK2SDALGHVLNMIGRDWTH
          	:**.* * ::*:**:*** * *:**: :.*:*********** *****************

MG01311.1 	AGVAKLYTDIQNNGYNIMYLTSRSVGQADTTRAYLHNIAQDGYRLPRGPTILSPDRTMAA
NCU03137.1	AGVAKLYTDIVANGYNIMYLTSRSVGQADTTRTYLAGIVQDGYRLPRGPTILSPDRTMAA
FG00866.1 	SGVAKLYSDISANGYNIMYLTSRSVGQSDTTRAYLAGIVQEGYKMPPGPTILSPDRTMAA
AN0802.1  	AGVAKLYTDIVNNGYNIMYLTSRSVGQADTTRSYIYGVCQDGYRLPKGPTIMSPDRTMAA
          	:******:**  ***************:****:*: .: *:**::* ****:********

MG01311.1 	LRREIYLRKPHVFKMATLRDIKSLYGLEHHPFYAGFGNRLTDQISYRTVDVPRTRIFTIN
NCU03137.1	LRREIYLRKPHIFKMSTLRDIRNLYGPDRTPFYAGFGNRFTDQISYRTVDVPRNRIFTIN
FG00866.1 	LRREVYLRKPHIFKMATLRDIRNLYGPDRTPFYAGYGNRLTDQISYRTVDVPRNRIFTIN
AN0802.1  	LRREIYLRKPEVFKMACLRDILGLFNGKENPFYAGFGNRLTDALSYRSVNIPSSRIFTIN
          	****:*****.:***: **** .*:. .. *****:***:** :***:*::* .******

MG01311.1 	SNAEVSLDLLSLNKLKLSYLSMSEIVEHYFPPVSTLVKGGGEEFTDFKYWRDQPLEIDEF
NCU03137.1	SNAEVSLDLLSLNKLKLSYVNMTEVVDHYFPPVTTLIKGGGEDYTDFKYWRDQPLDLDEF
FG00866.1 	SNSEVSLDLLTLNKLKMSYVNINEVVDHYFPPVSTLVKGGGEEYTDFTYWRDDPLAVADF
AN0802.1  	SNAEVQLDLLSLNKYKSSYVSMRELVDHFFPPVSLLVQAGGEEYTDFMYWRDVPRDFDDI
          	**:**.****:*** * **:.: *:*:*:****: *::.***::*** **** *  . ::

MG01311.1 	SASDSDEVD----RDDAE----DDEDDEESQYD-----------EAADAMGESYISRNSI
NCU03137.1	SASDTEDERSGGRKTSAP----AVVDDHESEYEED------DEEEVGDGLVDSYISRNSV
FG00866.1 	SASESDDGDDDEEDDNGPGAPTSEYGDDEEEYDDEDVEIDEDVDEIGEGLADSYISRASM
AN0802.1  	STTDSEDESQADEEEEVDNEYDGELSDDGSELDDD--------EIAEEELGASFVS-QAT
          	*::::::  .     .  .      .*. .: :..        .   : :  *::*  : 

MG01311.1 	D---EGSVYYEDG---------------------------QEMLEDSAVYE--QEDADAE
NCU03137.1	DDYEEGSVASEDGDYDAEDDGEDDGEDGEEEEGEYDEEAAPEHMVDSAMLDSVYVDADAD
FG00866.1 	DEFAEASSVLGGS--------------------------VDEEKLKASMTRDLKETYDED
AN0802.1  	DSYEEAETQSVAS-----------------------------------------------
          	*.  *..     .                                               

MG01311.1 	----DDDDDN------DEAMESEDEDTAGRGKS-------PAASTVNGKKNSARK-----
NCU03137.1	GVITDDEDAEGKKIVEEEEVDNEEEEDGGLGAEEEEGLRRAAADLEQAMKEQERKREPTE
FG00866.1 	----DEDDIY------EDGQEGHNEE--GEDAS--------TATPLGMKKAAELK-----
AN0802.1  	----DDEEDF------EDEEDDEGPEVAVRGKD---------------------------
          	    *:::        ::  :..  : .  . .                           

MG01311.1 	----------------DAVS------DVDAEVITGVKNLKVNGD~MHR------------
NCU03137.1	AQLQVPVEQQSRDSSSSSVSPKVIEPDADAEIITGIKDLTVEKE0AEEEEEEEEEKGWKR
FG00866.1 	-------------------------EDVLAQHITGVGHLSIEK-~---------------
AN0802.1  	--------------------------------------------~---------------
          	                                                            

MG01311.1 	-~-------------------------
NCU03137.1	V1MPGPNISKILEYSSRLDNNLLTHPA
FG00866.1 	-~-------------------------
AN0802.1  	-~-------------------------
          	
```
